# Palladium-immobilization on KIT-6 mesoporous silica magnetite nanoparticles as a stable nanocatalyst for cross-coupling and homo-coupling reactions†

**DOI:** 10.1039/d5na00058k

**Published:** 2025-03-31

**Authors:** Zahra Moradi, Mostafa Koolivand, Mohsen Nikoorazm, Arash Ghorbani-Choghamarani

**Affiliations:** a Department of Chemistry, Faculty of Science, Ilam University Ilam Iran m.nikorazm@ilam.ac.ir; b Department of Organic Chemistry, Bu-Ali Sina University 6517838683 Hamedan Iran

## Abstract

KIT-6 mesoporous silica-coated magnetite nanoparticles are organized large-pore nanoparticles that facilitate an environmentally friendly procedure for Ullmann, Suzuki–Miyaura, and Stille reactions. Various techniques, such as FTIR, SEM, TGA, VSM, XRD, BET, and AAS, were used to investigate and identify the synthesized Fe_3_O_4_@SiO_2_@KIT-6@IS-Pd^0^ catalyst. The magnetite nanoparticles could be easily separated from the reaction mixture simply by magnetic decantation, and they could be reused for several consecutive runs without seeing a significant decrease in catalytic activity.

## Abstract 2

KIT-6 mesoporous silica-coated magnetite nanoparticles are organized large-pore nanoparticles that facilitate an environmentally friendly procedure for Ullmann, Suzuki–Miyaura, and Stille reactions.

## Graphical abstract

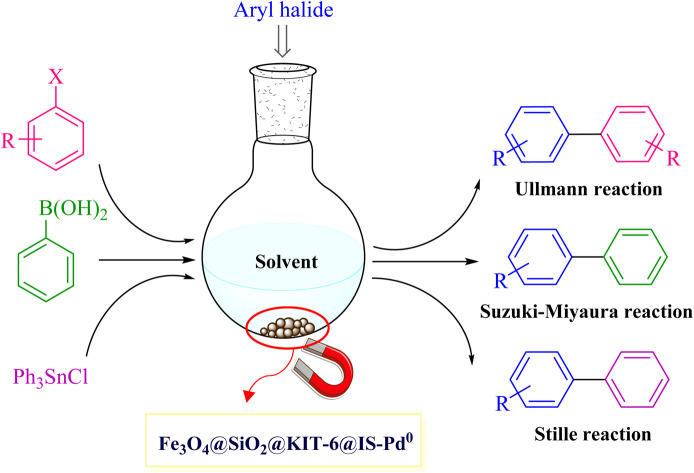

## Introduction

Green chemistry aims to decrease the use and manufacturing of dangerous chemicals for chemical reactions while lowering energy use and shifting toward renewable resources.^1,2^ In 1998, Anastas and Werner released the 12 principles of green chemistry to help researchers work toward this objective.^3–5^ Green chemistry is now seen as a means of presenting sustainable ideas at the fundamental level. According to its tenets, industrial and chemical process design should minimize the amount of end waste and avoid using toxic or hazardous solvents.^6,7^

Technological developments in organic chemistry cannot be envisioned without solvents. Organic solvents are carbon-based compounds that can dissolve or disperse one or more substances. In general, organic solvents are divided into two categories: polar and non-polar. In non-polar solvents like toluene, hexane, and benzene, which contain atoms with very similar electronegativities, the charge is symmetrically distributed across the molecules. Conversely, polar organic solvents (like ethanol, methanol, and acetonitrile) have larger dipole moments due to the different electronegativities of their atoms.^8^ Solvents have a variety of ways of affecting reactions. When a solute cannot be dissolved, solvents can be employed as a reactant to react with it, as a reaction medium to bring reactants together, and as a carrier to transport chemical compounds in solutions to their intended location in the necessary quantities.^9^

Carbon–carbon coupling reactions are one of the most important developments in chemical synthesis for preparing a wide range of organic compounds.^10,11^ They are a group of chemical reactions in organic chemistry in which a metal catalyst connects two hydrocarbon branches and forms a single structure.^12,13^ Carbon–carbon bond formation reactions can be used in various fields, including synthesizing natural and medicinal compounds, conducting polymers, sensors, dyes, insecticides, *etc.*^14–17^ C–C coupling reactions can be generally divided into homo-coupling reactions and cross-coupling reactions. C–C homo-coupling reactions are reactions in which two identical molecules pair together to form a symmetrical biaryl, such as the Ullmann reaction.^18,19^ C–C Cross-coupling reactions are reactions in which two different molecules form a new molecule, such as the Suzuki–Miyaura,^20^ Mizoroki–Heck,^21^ Stille,^22^ Sonogashira,^23^ Negishi,^24^ Kumada coupling,^25^ and Hiyama^26^ coupling reactions.

A composite is created by combining two or more different materials to give it properties superior to any constituent element. A nanocomposite is a multiphase solid substance with one, two, or three phases smaller than 100 nm in size or a structure in which the material's various phases have intermittent nanoscale distances. Nanocomposites primarily comprise several nanoparticles or nanomaterials combined with another bulk material. Nanocomposites could be organic–organic, inorganic–inorganic, or inorganic–organic phases. One such area where nanocomposites have become widely used is catalysis. The potential to create nanocomposites to enhance catalytic activity, selectivity, and stability makes them intriguing choices for various catalytic applications.^27–34^

Due to their unique properties, magnetic nanoparticles (MNPs) are extensively used in nanotechnology.^35^ Scientists can carefully design materials for specific needs by controlling size, morphology, and dispersion. The insoluble nature of magnetic materials allows for easy separation, which is one of the exciting properties of magnetic nanoparticles. This will enable nanocatalysts to be easily reused, two essential features of catalytic processes. Unlike heterogeneous catalytic systems that require filtration or solid separation for recovery, magnetic catalysts can be recycled without hard filtration steps. Additionally, the high potential for functionalizing separable magnetic catalysts makes them highly active in various reactions.^36,37^ On the other hand, KIT-6 has extremely uniform pores, a large number of silanol groups, and high stability. Therefore, porous silica KIT-6 supported with Fe_3_O_4_ MNPs has emerged as a powerful catalyst due to its small size, uniform porosity, high chemical stability, recyclability, and easy isolation.^38–40^ This study used Fe_3_O_4_ magnetic nanoparticles and KIT-6 porous silica to prepare the Fe_3_O_4_@SiO_2_@KIT-6@IS-Pd^0^ magnetic nanocatalyst. The prepared catalyst was used as an efficient, stable, and returnable catalyst for carbon–carbon coupling reactions. Fe_3_O_4_@SiO_2_@KIT-6@IS-Pd^0^ was robust for the Suzuki–Miyaura, Stille, and Ullmann reactions.

## Results and discussion

### Preparation of the Fe_3_O_4_@SiO_2_@KIT-6@IS-Pd^0^ magnetic catalyst

**Scheme 1:**
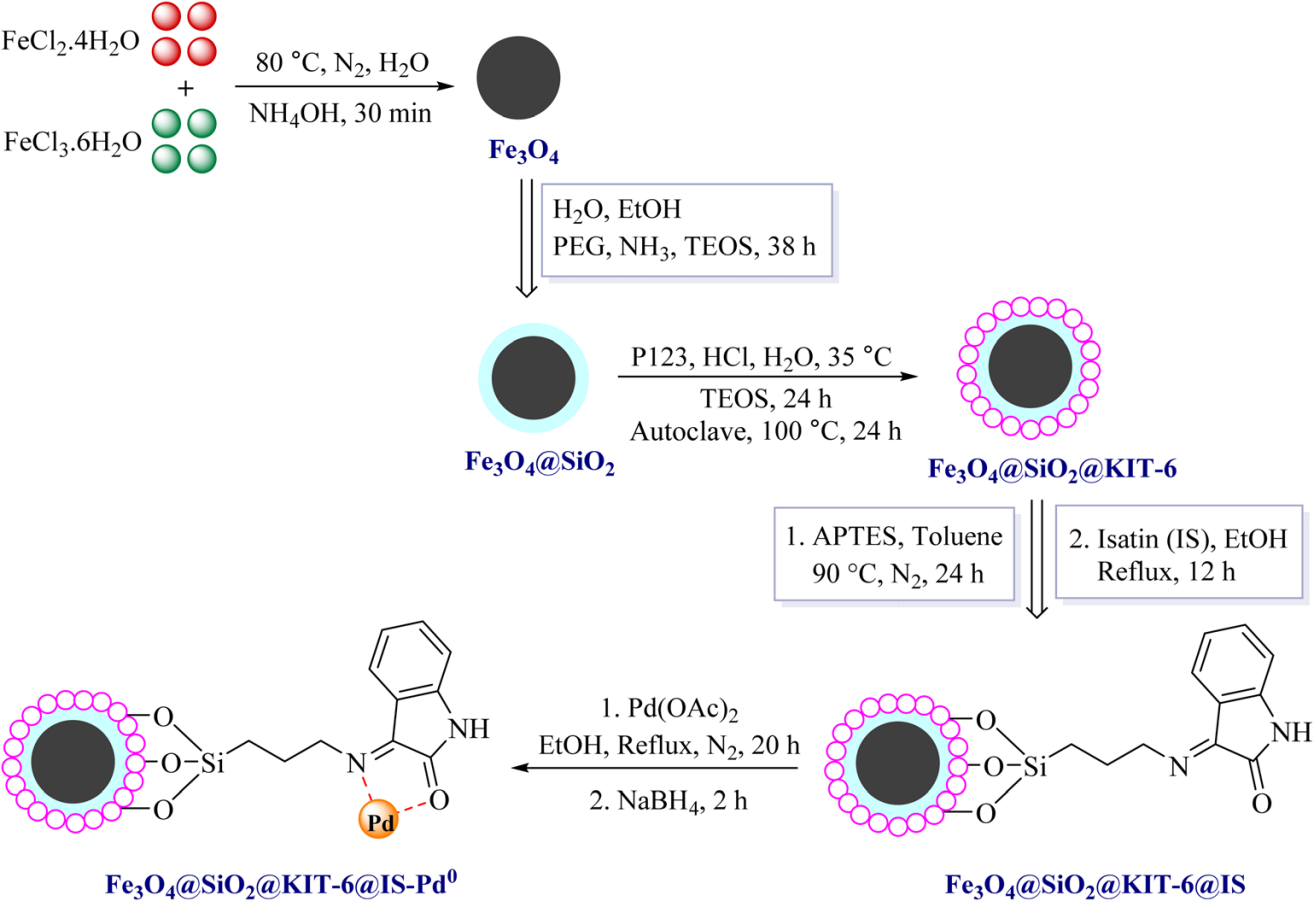
Formation steps of Fe_3_O_4_@SiO_2_@KIT-6@IS-Pd^0^.

We followed a series of steps to create the Fe_3_O_4_@SiO_2_@KIT-6@IS-Pd^0^ magnetic catalyst. Firstly, we synthesized the Fe_3_O_4_@SiO_2_@KIT-6 magnetic nanoparticles using previously reported methods. Then, we introduced functional groups to these nanoparticles using 3-aminopropyltriethoxysilane and isatin. Finally, we immobilized palladium metal on the Fe_3_O_4_@SiO_2_@KIT-6@IS nanocatalyst, as shown in Scheme 1.

This text briefly explains the process of designing and manufacturing the nanocatalyst. Fe_3_O_4_@SiO_2_@KIT-6@IS-Pd^0^ was identified using FTIR, SEM, TGA, VSM, XRD, BET, and AAS techniques.

**Fig. 1:**
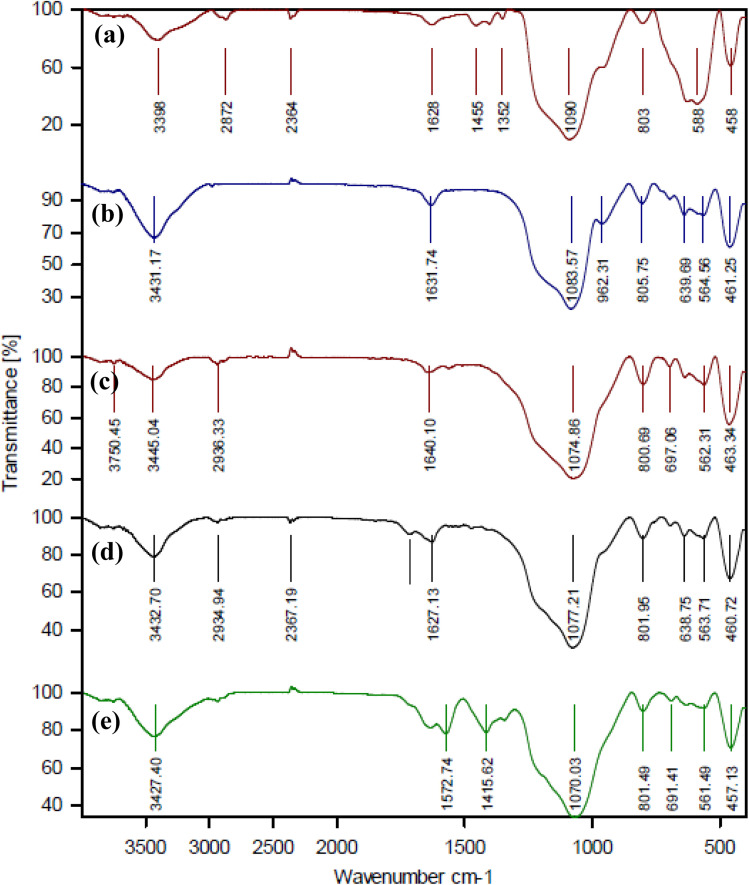
The FT-IR spectra of (a) Fe_3_O_4_@SiO_2_, (b) Fe_3_O_4_@SiO_2_@KIT-6, (c) Fe_3_O_4_@SiO_2_@KIT-6@APTES, (d) Fe_3_O_4_@SiO_2_@KIT-6@IS and (e) Fe_3_O_4_@SiO_2_@KIT-6@IS-Pd^0^.

Fig. 1 shows the FT-IR spectrum of the synthesis steps used to prepare the Fe_3_O_4_@SiO_2_@KIT-6@IS-Pd^0^ catalyst. The range of Fe_3_O_4_@SiO_2_ (Fig. 1a) shows peaks at 458 cm^−1^ and 588 cm^−1^, corresponding to the Fe–O vibration. The peak at 1090 cm^−1^ is related to the vibration of the Si–O–Si. The spectrum in Fig. 1b shows peaks at 1631 cm^−1^ and 3431 cm^−1^, which correspond to the bending and stretching of the OH group. In the spectrum of Fe_3_O_4_@SiO_2_@KIT-6@APTES (Fig. 1c), the 3389 and 2936 cm^−1^ peaks are related to the chain C–H and N–H stretching, respectively. The 1627 and 1721 cm^−1^ peaks in the FT-IR spectrum of Fe_3_O_4_@SiO_2_@KIT-6@IS (Fig. 1d) correspond to C=N and C=O stretching vibrational bands. The N–H stretching (the amide group) in isatin was observed at 3427 cm^−1^. The shifting of C=N and C=O peaks in the FT-IR spectra of Fe_3_O_4_@SiO_2_@KIT-6@IS-Pd^0^ (Fig. 1e) indicates that palladium was successfully coordinated to isatin.

**Fig. 2:**
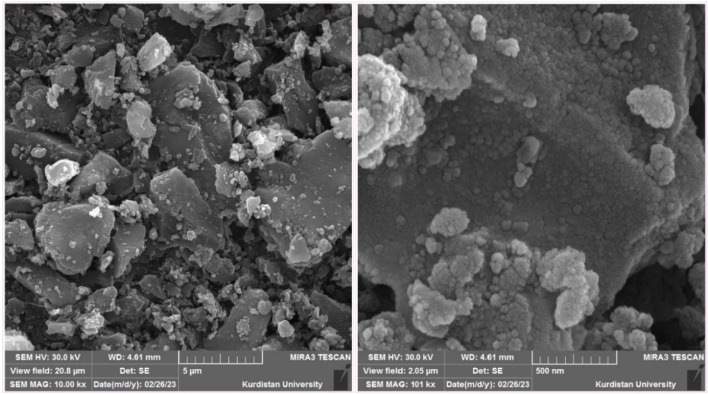
SEM image of the Fe_3_O_4_@SiO_2_@KIT-6@IS-Pd^0^ nanocatalyst.

To determine the morphology (shape) of the synthesized catalyst using a scanning electron microscope (SEM), high-magnification images of the Fe_3_O_4_@SiO_2_@KIT-6@IS-Pd^0^ nanocatalyst surface show spherical nanoparticles on the nanoscale. SEM images also showed spherical morphology of the synthesized nanocomposite (Fig. 2).^41^

**Fig. 3:**
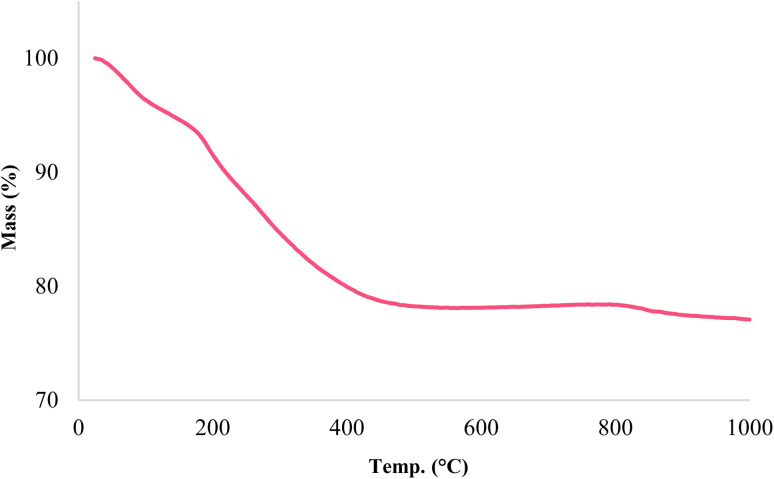
TGA diagram of the Fe_3_O_4_@SiO_2_@KIT-6@IS-Pd^0^ nanocatalyst.

The stability of catalyst Fe_3_O_4_@SiO_2_@KIT-6@IS-Pd^0^ was determined using the TGA technique. As shown in Fig. 3, the TGA diagram for the synthesized magnetic nanoparticles confirms that a weight loss of around 4% occurred at a temperature lower than 150 °C. This weight loss indicates the removal of the solvents absorbed on the catalyst surface. The second weight loss of about 16% was observed at a temperature between 100 and 500 °C, indicating the removal of organic materials stabilized on the magnetic nanoparticles.^41^

**Fig. 4:**
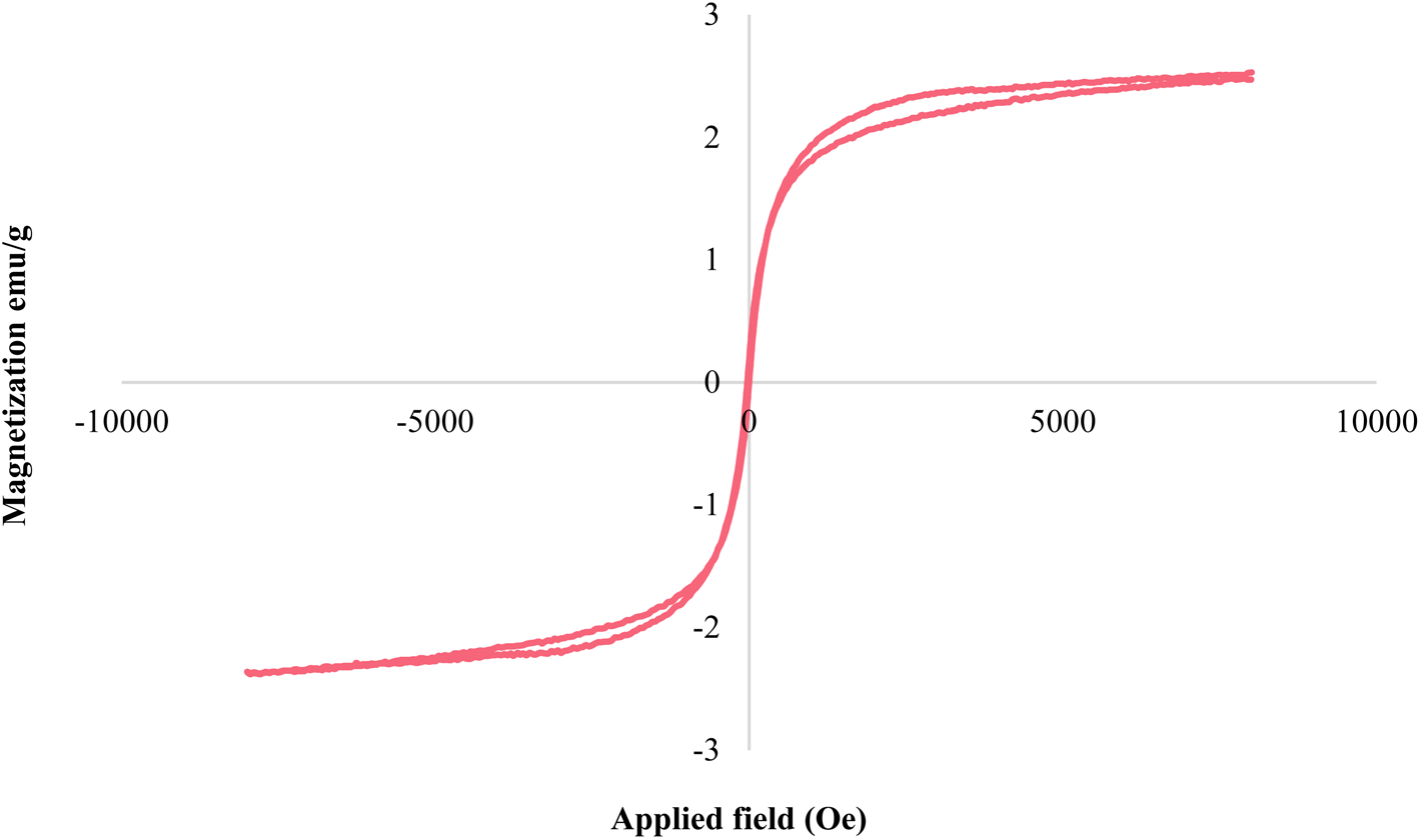
The magnetic curve of Fe_3_O_4_@SiO_2_@KIT-6@IS-Pd^0^.

Using VSM analysis, the magnetic properties of Fe_3_O_4_@SiO_2_@KIT-6@IS-Pd^0^ nanocatalysts were investigated. According to Fig. 4, the magnetic nature of the Fe_3_O_4_@SiO_2_@KIT-6@IS-Pd^0^ is 2.46 emu g^−1^, which means that the catalyst is functionalized with SiO_2_ and covered with organic groups. Finally, palladium metal is added to the nanocatalyst to stabilize it, resulting in good magnetic properties. However, an external magnet allows Fe_3_O_4_@SiO_2_@KIT-6@IS-Pd^0^ to be easily isolated from the reaction medium.^42^

**Fig. 5:**
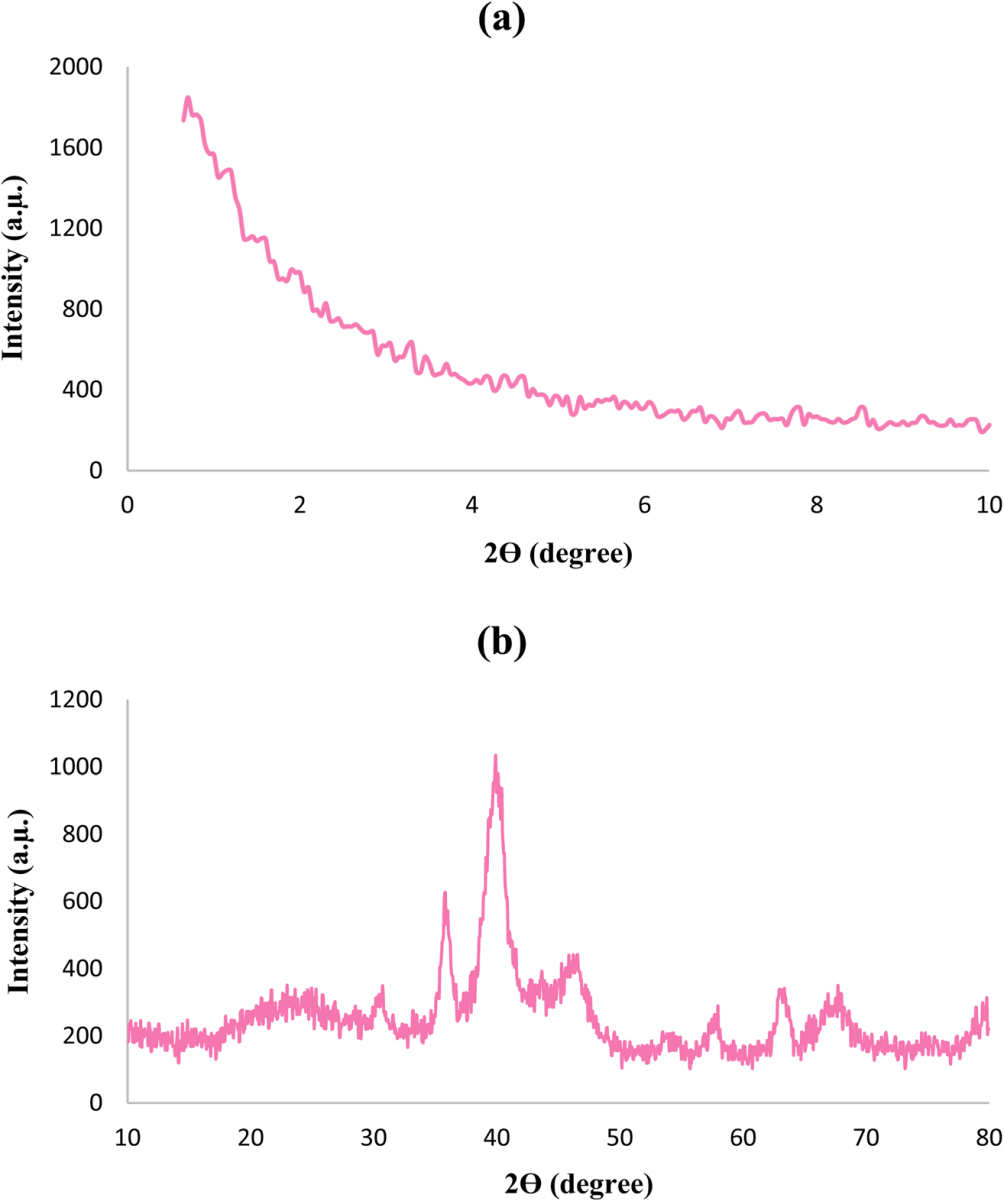
XRD patterns of the Fe_3_O_4_@SiO_2_@KIT-6@IS-Pd^0^ nanocatalyst; (a) XRD low angle, (b) XRD normal.

The XRD patterns of the Fe_3_O_4_@SiO_2_@KIT-6@IS-Pd^0^ nanocatalyst are presented in Fig. 5. As shown in the synthetic material's low-angle XRD pattern (Fig. 5a), KIT-6 shows a sharp diffraction peak at 2*Θ* = 0.8° and a series of weak peaks of KIT-6 at 2*Θ* = 1.2°–2°, corresponding to (211), (220), (420), and (332) crystal planes. The prepared KIT-6 has an ordered three-dimensional cubic mesoporous structure.^43,44^ As seen from the normal XRD pattern in Fig. 5b, the peaks at 24.36°, 30.26°, 35.76°, 42.76°, 52.56°, 57.66°, 61.62°, and 74.11° correspond to the peaks of Fe_3_O_4_ nanoparticles.^41,45^ The peak appearing at 21.66 is attrubuted to the presence of silica around the Fe_3_O_4_, and other peaks at 45.86° and 61.96° are indicative of palladium(0) metal-stabilized on the nanoparticles.^44^

**Fig. 6:**
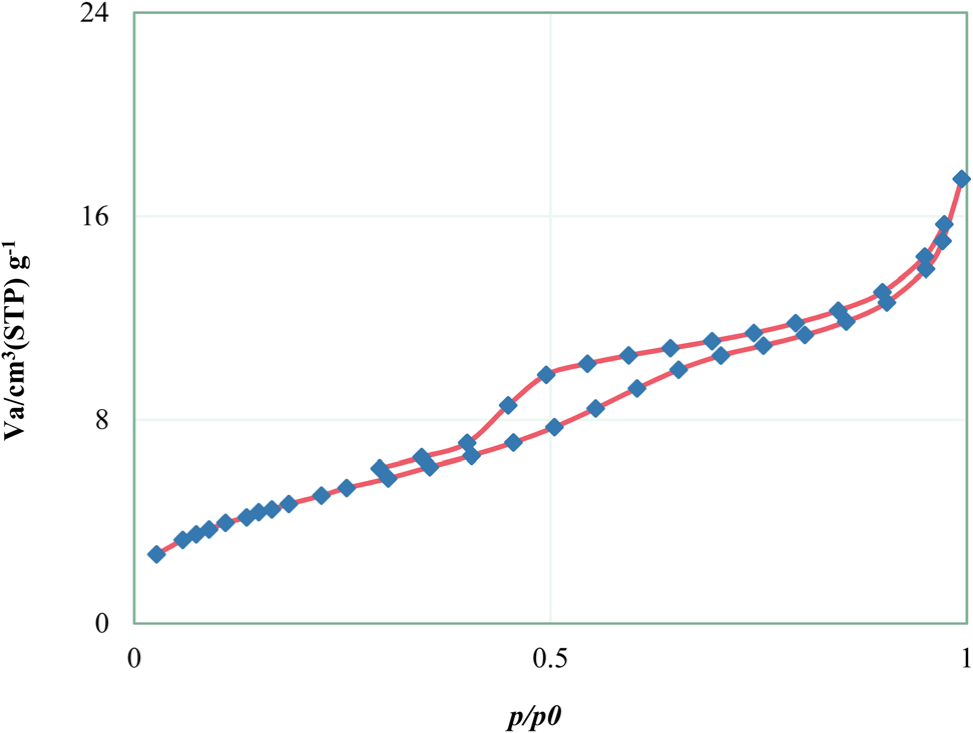
N_2_ adsorption–desorption isotherms of the Fe_3_O_4_@SiO_2_@KIT-6@IS-Pd^0^ nanocatalyst.

The nitrogen gas adsorption–desorption technique was used to determine the structural features and examine the surface of Fe_3_O_4_@SiO_2_@KIT-6@IS-Pd^0^ magnetic nanoparticles (Fig. 6). According to the IUPAC classification, the Fe_3_O_4_@SiO_2_@KIT-6@IS-Pd^0^ nanocatalyst shows a type IV isotherm characteristic of a mesoporous material. Based on the BET diagram, the surface area of the magnetic nanocatalyst is 17.417 m^2^ g^−1^, which shows that organic layers and the Pd complex are placed on the channels of Fe_3_O_4_@SiO_2_@KIT-6 magnetic nanoparticles. The calculations related to the BJH diagram give an average pore diameter of 2.13 nm.

Atomic absorption spectrometry (AAS) was used to obtain the exact amount of palladium in the catalyst. The precise content of stabilized palladium equals 1.16 × 10^−3^ mol g^−1^.

### Catalytic study

Studying is essential to obtaining the best conditions to perform a reaction. For this reason, to obtain the ideal conditions for the Ullmann coupling, the reaction of iodobenzene was first inspected in the presence of different amounts of Fe_3_O_4_@SiO_2_@KIT-6@IS-Pd^0^, various types of solvents, and bases at different temperatures (Table 1). In the first step, the optimization of the reaction of 2 mmol of iodobenzene in the presence of 4 mmol of K_2_CO_3_ and 5 mg of catalyst at a temperature of 130 °C in various solvents such as DMF, DMSO, and PEG was done (Entries 1–3). According to Table 1, PEG was chosen as the optimal reaction solvent because the reaction efficiency was high in this solvent. In the next step, the amount of catalyst was optimized, and according to Table 1, the best reaction results were obtained when the amount of catalyst was 5 mg. In the end, the reaction temperature was checked, and according to the optimization table, the reaction efficiency also decreased with the decrease in the reaction temperature. As a result, PEG solvent, 4 mmol of K_2_CO_3_, 5 mg of catalyst, and a temperature of 130 °C were chosen as ideal conditions for this reaction.

**Table 1:** Experimental optimization conditions for the Ullmann reaction in the presence of Fe_3_O_4_@SiO_2_@KIT-6@IS-Pd^0^

Entry	Solvent	Base	Base (mmol)	Catalyst (mg)	Temperature (°C)	Time (min)	Yield^a^ (%)
1	DMSO	K_2_CO_3_	4	5	130	90	66
2	DMF	K_2_CO_3_	4	5	130	90	62
3	**PEG**	**K**_**2**_ **CO**_**3**_	**4**	**5**	**130**	**90**	**91**
4	PEG	K_2_CO_3_	5	5	130	90	91
5	PEG	K_2_CO_3_	4	6	130	90	92
6	PEG	K_2_CO_3_	4	4	130	90	83
7	PEG	K_2_CO_3_	4	5	110	90	71
8	PEG	K_2_CO_3_	4	5	100	90	61

a Isolated yield.

After obtaining the optimal conditions, more products of the Ullmann coupling were synthesized using different aryl halides in good yields (Table 2).

**Table 2:** Ullmann reaction in the presence of Fe_3_O_4_@SiO_2_@KIT-6@IS-Pd^0^

Entry	X	R	Time (min)	Yield (%)	TON	TOF (h^−1^)	M.P (°C)
1	I	H	90	91	156.9	104.6	68–69 (ref. 46)
2	I	4-CH_3_	90	87	150	100	123–124 (ref. 47)
3	I	4-OCH_3_	90	88	151.7	101.1	171–172 (ref. 48)
4	Br	4-OCH_3_	90	79	136.2	90.8	170–172 (ref. 48)
5	Br	4-CH_3_	90	74	127.5	85.0	122–123 (ref. 49)
6	Br	H	90	82	141.3	94.2	67–68 (ref. 46)
7	Br	4-Cl	90	70	120.9	80.6	144–146 (ref. 47)

### Proposed mechanism for the synthesis of symmetrical biphenyl derivatives using Ullmann reaction

**Scheme 2:**
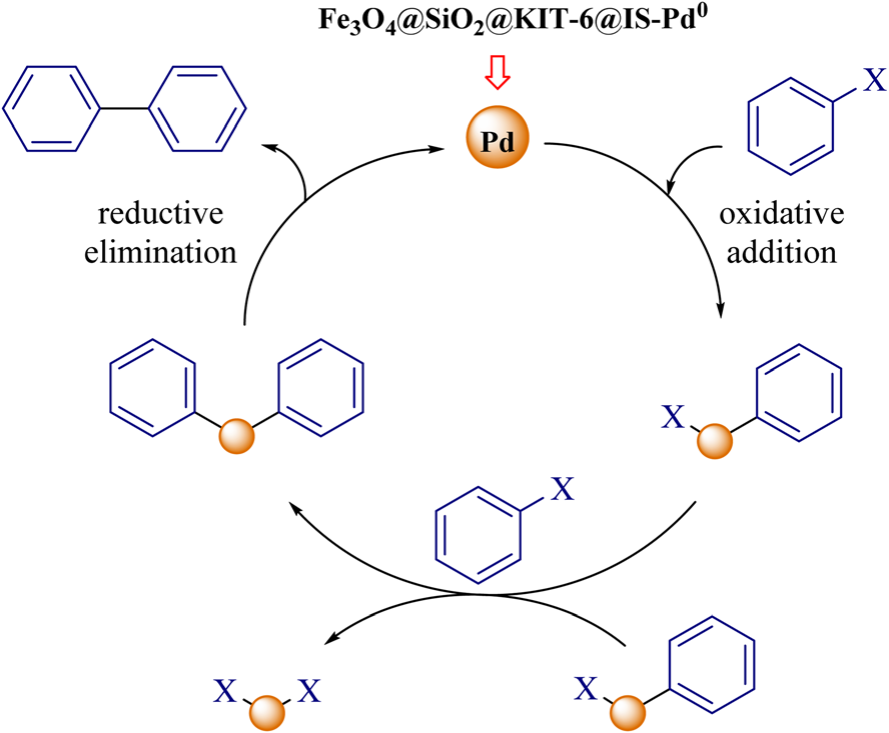
A possible mechanism for the Ullmann reaction by using the Fe_3_O_4_@SiO_2_@KIT-6@IS-Pd^0^ nanocatalyst.

Scheme 2 shows the reaction mechanism of the Ullmann reaction with the Fe_3_O_4_@SiO_2_@KIT-6@IS-Pd^0^ nanocatalyst.^50^

### Suzuki–Miyaura reaction

To optimize the Suzuki–Miyaura reaction conditions, iodobenzene with phenylboronic acid was selected as a sample reaction. The optimization parameters were investigated in this reaction. The effects of base, temperature, solvent, and catalyst amount were analyzed (Table 3). The reaction was investigated in different solvents, including polyethylene glycol, ethanol, acetonitrile, dimethyl sulfoxide, and dimethyl formamide. The results showed that ethanol provides the best environment for performing the reaction with higher efficiency. Then, the catalyst optimization was done, and 5 mg of catalyst was considered the optimal amount. Different bases were also investigated for this reaction, and potassium carbonate had the best efficiency. Finally, three mmol of potassium carbonate, ethanol solvent, 5 mg of catalysts, and a temperature of 70 °C were selected as optimal conditions for the Suzuki–Miyaura reaction.

**Table 3:** Experimental optimization of Suzuki coupling of phenylboronic acid and iodobenzene

Entry	Solvent	Base	Base (mmol)	Catalyst (mg)	Temperature (°C)	Time (min)	Yield^a^ (%)
1	DMSO	K_2_CO_3_	3	5	70	30	75
2	DMF	K_2_CO_3_	3	5	70	30	70
3	CH_3_CN	K_2_CO_3_	3	5	70	30	53
4	PEG	K_2_CO_3_	3	5	70	30	92
5	**EtOH**	**K**_**2**_ **CO**_**3**_	**3**	**5**	**70**	**30**	**96**
6	EtOH	K_2_CO_3_	3	4	70	30	76
7	EtOH	K_2_CO_3_	3	6	70	25	96
8	EtOH	Na_2_CO_3_	3	5	70	30	71
9	EtOH	NaOH	3	5	70	30	65
10	EtOH	KOH	3	5	70	30	76
11	EtOH	K_2_CO_3_	1.5	5	70	30	46
12	EtOH	K_2_CO_3_	3	5	60	30	69
13	EtOH	K_2_CO_3_	3	5	40	30	43

a Isolated yield.

After obtaining the optimal conditions, the reaction was investigated for different types of aryl halides to expand this catalyst's application range in the synthesis of biaryl compounds. The results are shown in Table 4.

**Table 4:** Suzuki coupling in the presence of Fe_3_O_4_@SiO_2_@KIT-6@IS-Pd^0^

Entry	X	R	Time (min)	Yield (%)	TON	TOF (h^−1^)	M.P (°C)
1	I	H	30	96	165.5	331	69–70 (ref. 51)
2	I	4-CH_3_	24	95	163.8	409.5	47–48 (ref. 52)
3	I	4-OCH_3_	20	96	165.5	496.5	88–89 (ref. 53)
4	Br	4-OCH_3_	65	94	162.1	149.6	87–89 (ref. 54)
5	Br	4-CH_3_	75	90	155.2	124.1	46–48 (ref. 52)
6	Br	4-OH	70	92	158.6	135.9	162–165 (ref. 55)
7	Br	3-OCH_3_	60	94	162.1	162.1	89–90 (ref. 56)
8	Br	H	45	89	153.4	204.5	69–70 (ref. 56)
9	Br	4-Cl	80	87	150	112.5	68–70 (ref. 51)
10	Br	4-NO_2_	100	88	151.7	91.0	112–114 (ref. 56)
11	Cl	H	180	85	146.5	48.8	66–68 (ref. 51)

### Stille reaction

To obtain the ideal conditions, the reaction between iodobenzene and triphenyl tin chloride in the presence of the Fe_3_O_4_@SiO_2_@KIT-6@IS-Pd^0^ nanocatalyst was selected as a sample reaction. Then, various parameters, including the type of base, amount of catalyst, nature of solvent, and temperature, were investigated (results are summarized in Table 5). First, the reaction was tested in several solvents, such as DMSO, DMF, EtOH, and PEG, and the best result was obtained in PEG (Entry 2). Next, the amount of catalyst was investigated; according to the reaction optimization table, the efficiency is excellent in the presence of 5 mg of Fe_3_O_4_@SiO_2_@KIT-6@IS-Pd^0^. Then, reaction progress was examined among different bases (such as K_2_CO_3_, Na_2_CO_3_, NaOH, and KOH), where potassium carbonate was selected as the optimal base for the reaction. According to Table 5 Entry 11, the reaction efficiency decreased significantly with decreasing the amount of potassium carbonate. To optimize the reaction temperature, the reaction was checked at 60 °C, 80 °C and 100 °C, and the results showed that the suitable temperature for the reaction was 80 °C.

**Table 5:** Experimental optimization study of Stille coupling of triphenyl tin chloride and iodobenzene

Entry	Solvent	Base	Base (mmol)	Catalyst (mg)	Temperature (°C)	Time (min)	Yield^a^ (%)
1	EtOH	K_2_CO_3_	3	5	80	35	58
2	**PEG**	**K**_**2**_ **CO**_**3**_	**3**	**5**	**80**	**35**	**95**
3	DMSO	K_2_CO_3_	3	5	80	35	56
4	DMF	K_2_CO_3_	3	5	80	35	57
5	PEG	K_2_CO_3_	3	4	80	35	81
6	PEG	K_2_CO_3_	3	6	80	35	95
7	PEG	K_2_CO_3_	3	7	80	30	93
8	PEG	Na_2_CO_3_	3	5	80	35	84
9	PEG	KOH	3	5	80	35	87
10	PEG	NaOH	3	5	80	35	79
11	PEG	K_2_CO_3_	1.5	5	80	35	49
12	PEG	K_2_CO_3_	3	5	60	35	36
13	PEG	K_2_CO_3_	3	5	100	32	94

a Isolated yield.

As shown in Table 6, many biphenyls were obtained from the reaction of aryl halides with triphenyl tin chloride in the presence of the Fe_3_O_4_@SiO_2_@KIT-6@IS-Pd^0^ catalyst under optimal reaction conditions with good to excellent yields. Great breadth and diversity were seen in the synthesis of biaryl in terms of the type and position of the substituents on the aryl groups.

**Table 6:** The Stille reaction by Fe_3_O_4_@SiO_2_@KIT-6@IS-Pd^0^

Entry	X	R	Time (min)	Yield (%)	TON	TOF (h^−1^)	M.P (°C)
1	I	H	35	95	167.3	200.7	67–69 (ref. 42)
2	I	4-CH_3_	33	94	162.1	294.7	44–46 (ref. 57)
3	I	4-OCH_3_	30	92	158.6	317.2	86–87 (ref. 58)
4	Br	4-OCH_3_	70	92	158.6	135.9	86–8 (ref. 58)
5	Br	4-CH_3_	85	90	155.1	109.5	43–45 (ref. 57)
6	Br	4-OH	65	89	153.4	141.6	161–163 (ref. 59)
7	Br	3-OCH_3_	85	87	150	105.9	87–88 (ref. 42)
8	Br	H	100	89	153.4	92.0	68–70 (ref. 42)
9	Br	4-NO_2_	180	83	143.1	47.7	112–114 (ref. 59)
10	Br	4-Cl	120	85	146.5	73.2	69–70 (ref. 42)
11	Cl	H	280	80	137.9	103.4	67–69 (ref. 42)

### Proposed mechanism for the synthesis of biaryl derivatives using the carbon–carbon cross-coupling reaction

**Scheme 3:**
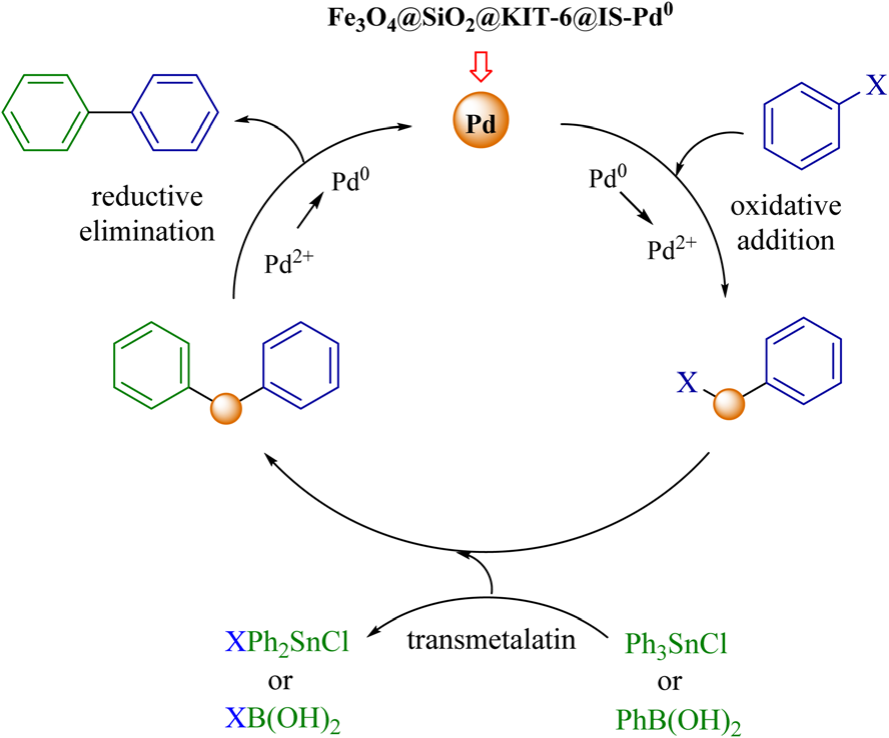
A possible mechanism for the Suzuki–Miyaura and Stille reactions by using the Fe_3_O_4_@SiO_2_@KIT-6@IS-Pd^0^ nanocatalyst.

The following mechanism can be proposed for synthesizing biaryl derivatives to perform Suzuki and Stille reactions in the presence of the palladium magnetic nanocatalyst (Scheme 3).^60^

### Catalyst recovery

**Fig. 7:**
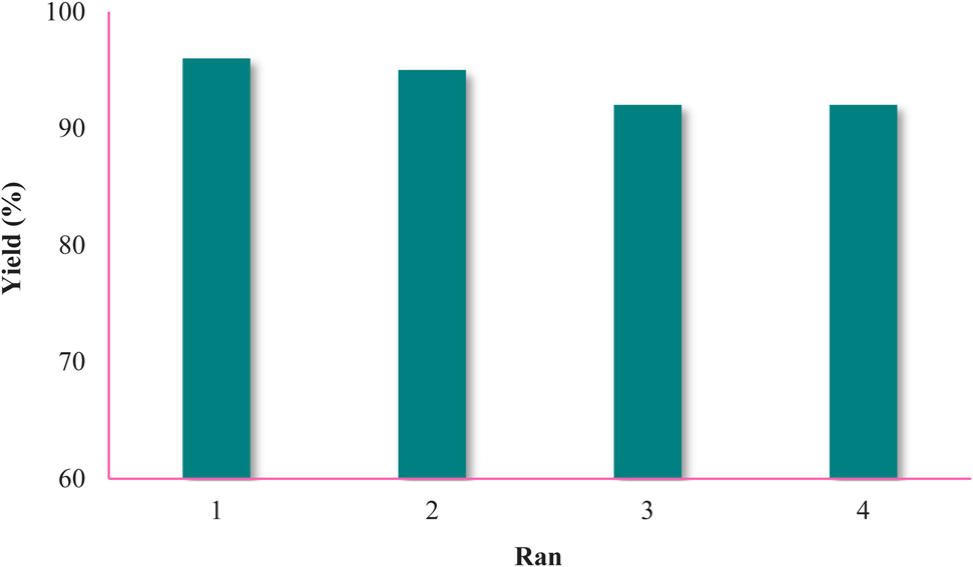
Recycling and reuse of the Fe_3_O_4_@SiO_2_@KIT-6@IS-Pd^0^ nanocatalyst.

To check the recycling of Fe_3_O_4_@SiO_2_@KIT-6@IS-Pd^0^, according to the general synthesis method of the Suzuki coupling, the reaction of iodobenzene (1 mmol, 0.204 mg), phenylboronic acid (1 mmol, 0.121 mg), Fe_3_O_4_@SiO_2_@KIT-6@IS-Pd^0^ (5 mg), and K_2_CO_3_ (3 mmol, 0.414 mg) was chosen as a sample reaction in ethanol solvent and the mixing took place at 70 °C for a defined period. After the end of each reaction, Fe_3_O_4_@SiO_2_@KIT-6@IS-Pd^0^ was separated by applying a magnetic field; after washing the catalyst with ethanol and drying it, it was used for the further step. As seen in Fig. 7, Fe_3_O_4_@SiO_2_@KIT-6@IS-Pd^0^ can be recycled and reused up to 4 times without reducing the catalytic activity in this reaction.

## Experimental

### Materials and devices

All chemicals purchased from Sigma and Merck companies were used without purification. All the products are known, which were confirmed by comparing the physical and spectral data reported in the articles, and the melting point was determined with an Electrothermal 9100 apparatus.

## Preparation of Fe_3_O_4_@SiO_2_@KIT-6@IS-Pd^0^

### Preparation of Fe_3_O_4_@SiO_2_@KIT-6@APTES

To synthesize Fe_3_O_4_@SiO_2_@KIT-6@APTES, Fe_3_O_4_@SiO_2_@KIT-6 was first prepared by a method erstwhile published in the literature.^41,42^ Then, 1 g of Fe_3_O_4_@SiO_2_@KIT-6 was sonicated in 20 mL of toluene for 15 minutes, then 1.5 mL of (3-aminopropyl) triethoxysilane (APTES) was added, and further the mixture was stirred for 24 h (at 90 °C). The powder was washed three times with CH_2_Cl_2_ and dried.^61^

### Preparation of Fe_3_O_4_@SiO_2_@KIT-6@IS

In this step, an ultrasonic device dispersed 1 g of Fe_3_O_4_@SiO_2_@KIT-6@APTES nanoparticles in 20 mL of ethanol for 10 minutes. Then, 2 mmol (0.294 mg) of isatin (IS) was injected into the mixture and further stirred under reflux for 12 h. The synthesized magnetic nanoparticles were separated with a magnetic magnet, washed with water and ethanol four times, and dried at room temperature.^62^

### Preparation of Fe_3_O_4_@SiO_2_@KIT-6@IS-Pd^0^

To prepare Fe_3_O_4_@SiO_2_@KIT-6@IS-Pd^0^, 1 g of magnetic nanoparticles functionalized with isatin was dispersed in 20 mL of ethanol solvent for 20 min, then Pd(OAc)_2_ (0.5 g) was injected under reflux conditions and stirred for 20 hours. Then 0.6 mmol of NaBH_4_ (0.022 mg) was added and stirred for 2 hours under the same conditions. After cooling, the reaction mixture was separated with a magnet, washed several times with ethanol, and dried.^42^

### General method Ullmann reaction by the Fe_3_O_4_@SiO_2_@KIT-6@IS-Pd^0^ nanocatalyst

To perform the Ullmann reaction, aryl halide (2 mmol), K_2_CO_3_ (4 mmol) and 5 mg of Fe_3_O_4_@SiO_2_@KIT-6@IS-Pd^0^ catalyst were added to 2 mL of polyethylene glycol (PEG) and further stirred at 130 °C. TLC followed the progress of the reaction in the *n*-hexane solvent until the reaction was complete. Then, the nanoparticles and inorganic materials were separated by a magnet using water and ethyl acetate and subjected to spectral analysis after purification.
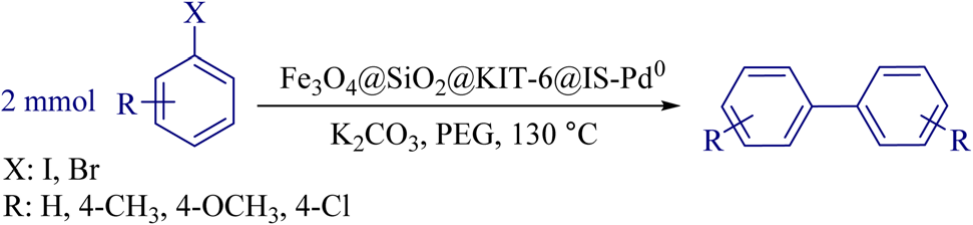


### General method Suzuki–Miyaura reaction by the Fe_3_O_4_@SiO_2_@KIT-6@IS-Pd^0^ catalyst

One mmol of each aryl halide, K_2_CO_3_ as a base (3 mmol, 0.414 mg), Fe_3_O_4_@SiO_2_@KIT-6@IS-Pd^0^ (5 mg), and phenylboronic acid (1 mmol, 0.121 mg) were added to 2 mL of EtOH, and further, the mixture was stirred at 70 °C for a specified period. After the end of the reaction, an external magnetic field separated the catalyst. The reaction mixture was moved to a separator funnel and then extracted with ethyl acetate. The products were subjected to spectral analysis after purification.
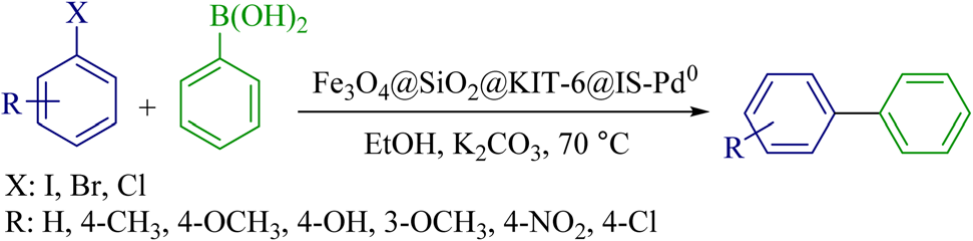


### General method Stille reaction by the Fe_3_O_4_@SiO_2_@KIT-6@IS-Pd^0^ catalyst

One mmol of each aryl halide, Fe_3_O_4_@SiO_2_@KIT-6@IS-Pd^0^ (5 mg), triphenyl tin chloride (0.5 mmol, 0.192 mg), and K_2_CO_3_ as a base (3 mmol, 0.414 mg) were added to 2 mL of polyethylene glycol (PEG) solvent, and the reaction mixture was stirred at 80 °C. After the completion of each reaction, Fe_3_O_4_@SiO_2_@KIT-6@IS-Pd^0^ was removed by an external magnetic field, and the reaction mixture was moved to a separatory funnel and further extracted with ethyl acetate; then, the obtained product was subjected to spectral analysis after purification.
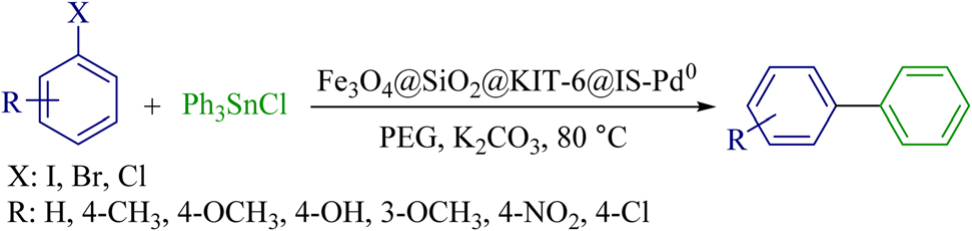


### Selected spectral data

#### 4,4-Dimethyl biphenyl

Mp: 123–124 °C; TLC (*n*-hexane); ^1^H NMR (500 MHz, CDCl_3_) *δ* = 7.52–7.51 (d, 4H), 7.25–7.23 (d, 4H), 2.33 (s, 6H, CH_3_) ppm (FS 1). ^13^CNMR (125 MHz, CDCl_3_) *δ* = 137.23, 136.35, 129.50, 126.35, 20.64 ppm (FS 5).^49^

#### 4-Methyl biphenyl

Mp: 46–48 °C; TLC (*n*-hexane); ^1^H NMR (500 MHz, CDCl_3_) *δ* = 7.64–7.62 (d, 2H), 7.56–7.54 (d, 2H), 7.46–7.43 (d, 2H), 7.33–7.32 (t, 1H), 7.27–7.26 (d, 2H), 2.34 (s, 3H, CH_3_) ppm (FS 2).^42^

#### 4-Methoxy biphenyl

Mp: 46–48 °C; TLC (*n*-hexane); ^1^H NMR (500 MHz, CDCl_3_) *δ* = 7.64–7.62 (d, 2H), 7.56–7.54 (d, 2H), 7.46–7.43 (d, 2H), 7.33–7.32 (t, 1H), 7.27–7.26 (d, 2H), 2.34 (s, 3H, CH_3_) ppm (FS 3).^63^

#### 4-Nitro biphenyl

Mp: 113–114 °C; TLC (*n*-hexane); ^1^H NMR (500 MHz, CDCl_3_) *δ* 8.32–8.29 (d, 2H), 7.76–7.73 (d, 2H), 7.65–7.63 (d, 2H), 7.54–7.51 (d, 2H), 7.49–7.48 (t, 1H) ppm (FS 4).^42^

### Comparison of the efficiency of catalyst Fe_3_O_4_@SiO_2_@KIT-6@IS-Pd^0^ in contrast with previous catalysts in the Suzuki–Miyaura reaction

This synthetic method has been compared with other presented methods to compare the catalytic activity of Fe_3_O_4_@SiO_2_@KIT-6@IS-Pd^0^ with other catalysts used to synthesize biaryls. As shown in Table 7, in this method, ethanol solvent was used to prepare biphenyl (reaction of 4-iodotoluene with phenylboronic acid), and the product was obtained in less time and with higher efficiency.

**Table 7:** Comparison of the efficiency of catalyst Fe_3_O_4_@SiO_2_@KIT-6@IS-Pd^0^ in contrast with previous catalysts in the Suzuki–Miyaura reaction

Entry	Catalyst	Conditions	Time (h)	Yield (%)	Ref.
1	Pd NPs/RGO (1 mol%)	EtOH/H_2_O, K_2_CO_3_, 50 °C	1	95	64
2	MCNTs@(A-V)-silica-Pd (1.5 mol%)	EtOH, CsCO_3_, reflux	8	93	65
3	GO-Met-Pd (0.1 mol%)	EtOH/H_2_O, K_2_CO_3_, 60 °C	0.25	98	66
4	Fe_3_O_4_@CS@MS@Pd (0.1 mol%)	EtOH/H_2_O, K_2_CO_3_, 70 °C	1	96	67
5	Pd NPs@P(3-MPAP) (0.007 mol%)	MW, 400 W	0.083	81	68
6	Pd NPs@CS/δ-FeOOH (0.05 mol%)	EtOH/H_2_O, K_2_CO_3_, 70 °C	3	90	69
7	Pd-SBTU@Fe_3_O_4_@SBA-3 (5 mg)	EtOH, K_2_CO_3_, 70 °C	1	94	70
8	MMCM-41@APy-Pd (7 mg)	PEG-200, Na_2_CO_3_, 80 °C	0.66	95	71
9	Fe_3_O_4_@SiO_2_-Pd (10 mg)	EtOH/H_2_O, K_2_CO_3_, 90 °C	1.5	92	72
10	Fe_3_O_4_@SiO_2_@KIT-6@IS-Pd^0^ (5 mg)	EtOH, K_2_CO_3_, 70 °C	0.4	95	This work

## Conclusion

Chemists try to use solvents, catalysts, and raw materials that provide the principles of green chemistry. In conclusion, we have examined the KIT-6 mesoporous silica-coated magnetite nanoparticles as a mild and efficient catalyst for biaryl synthesis under green conditions. The present procedure provides good turnover numbers (TON) and turnover frequency (TOF) in all reactions. The catalyst was reused for up to four cycles for the Suzuki–Miyaura reaction. The notable features of this catalytic procedure are the simplicity, easy workup, and use of a reusable, eco-friendly catalyst.

## Abbreviations

KIT-6: Korean Institute of Technology
KOH: Potassium hydroxide
P123: Poly(ethylene glycol)-*block*-poly(propylene glycol)-*block*-poly(ethylene glycol)
NaOH: Sodium hydroxide
IS: Isatin
nm: Nanometer
TEOS: Tetraethyl orthosilicate
M.P: Melting point
APTES: (3-Aminopropyl)triethoxysilane
FT-IR: Fourier transform infrared
PEG: Polyethylene glycol
SEM: Scanning electron microscopy
EtOH: Ethanol
TGA: Thermogravimetric analysis
DMF: Dimethylformamide
VSM: Vibrating sample magnetometer
DMSO: Dimethyl sulfoxide
XRD: X-ray diffraction
K_2_CO_3_: Potassium carbonate
BET: Brunauer–Emmett–Teller
Na_2_CO_3_: Sodium carbonate
AAS: Atomic absorption spectrometry

## Ethical approval

This article does not include experiments involving human tissue.

## Data availability

All data are available in the article and the ESI.†

## Author contributions

Zahra Moradi: experimental work, methodology, software, writing, review, and editing. Mostafa Koolivand: investigation and data curation. Mohsen Nikoorazm: supervised the research project, writing, review, and editing. Arash Ghorbani-Choghamarani: supervised the research project.

## Conflicts of interest

All co-authors have seen and agreed with the manuscript's contents, and there are no conflicts of interest or competing interests.

## Supplementary Material

NA-OLF-D5NA00058K-s001
