# Supplementary material for: Palladium-immobilization on KIT-6 mesoporous silica magnetite nanoparticles as a stable nanocatalyst for cross-coupling and homo-coupling reactions
Source: Nanoscale Adv. 2025 Mar 31;7(10):3114–24. doi: 10.1039/d5na00058k (PMC11979489; doi:10.1039/d5na00058k)
Supplement: NA-007-D5NA00058K-s001 [file NA-007-D5NA00058K-s001.pdf]

Supplementary Information (SI) for Nanoscale Advances.
This journal is © The Royal Society of Chemistry 2025

Palladium-immobilization on KIT-6 mesoporous silica magnetite nanoparticles as stable nanocatalyst for cross-coupling and homo-coupling reactions

Zahra Moradi [a], Mostafa Koolivand [a], Mohsen Nikoorazm [a*], Arash Ghorbani-Choghamarani [b]

^{1}HNMR and ^{13}CNMR spectrum of biphenyl compounds synthesized with nanocatalyst Fe_3O_4@SiO_2@KIT-6@IS-Pd^0

1. Homo-coupling reaction (Ullmann reaction)

4,4-dimethyl biphenyl

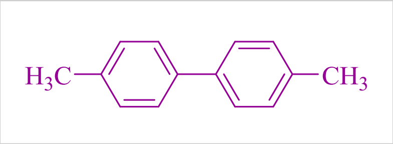

mp: 123–124 °C; TLC (n-hexane); ^{1}H NMR (300 MHz, $CDCl_3$) δ= 7.52-7.51 (d, 4H), 7.25-7.23 (d, 4H), 2.33 (s, 6H, CH_3) ppm (**Figure 1**).

mp: 123–124 °C; TLC (n-hexane); ^{13}CNMR (100 MHz, CDCl3) δ = 137.23, 136.35, 129.50, 126.35, 20.64 ppm (**Figure 5**).

---

2. Cross-coupling reaction (Suzuki-Miyaura, and Stille reactions)

4-Methyl biphenyl:

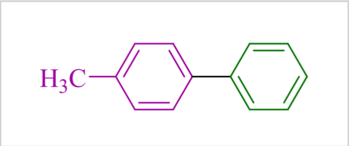

mp: 46–48 °C; TLC (n-hexane); ^{1}H NMR (300 MHz, $CDCl_3$) δ= 7.64-7.62 (d, 2H), 7.56-7.54 (d, 2H), 7.46-7.43 (d, 2H), 7.33-7.32 (t, 1H), 7.27-7.26 (d, 2H), 2.34 (s, 3H, CH_3) ppm (**Figure 2**).

4-Methoxy biphenyl:

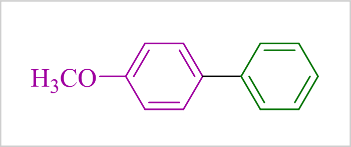

mp: 46–48 °C; TLC (n-hexane); ^{1}H NMR (300 MHz, $CDCl_3$) δ= 7.64-7.62 (d, 2H), 7.56-7.54 (d, 2H), 7.46-7.43 (d, 2H), 7.33-7.32 (t, 1H), 7.27-7.26 (d, 2H), 2.34 (s, 3H, CH_3) ppm (**Figure 3**).

4-Nitro biphenyl:

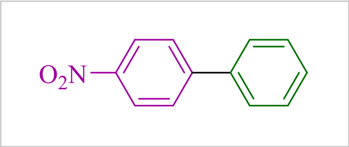

mp: 113-114 °C; TLC (n-hexane); ^{1}H NMR (300 MHz, $CDCl_3$) δ 8.32-8.29 (d, 2H), 7.76-7.73 (d, 2H), 7.65-7.63 (d, 2H), 7.54-7.51 (d, 2H), 7.49-7.48 (t, 1H) ppm (**Figure 4**).

^{1}HNMR:

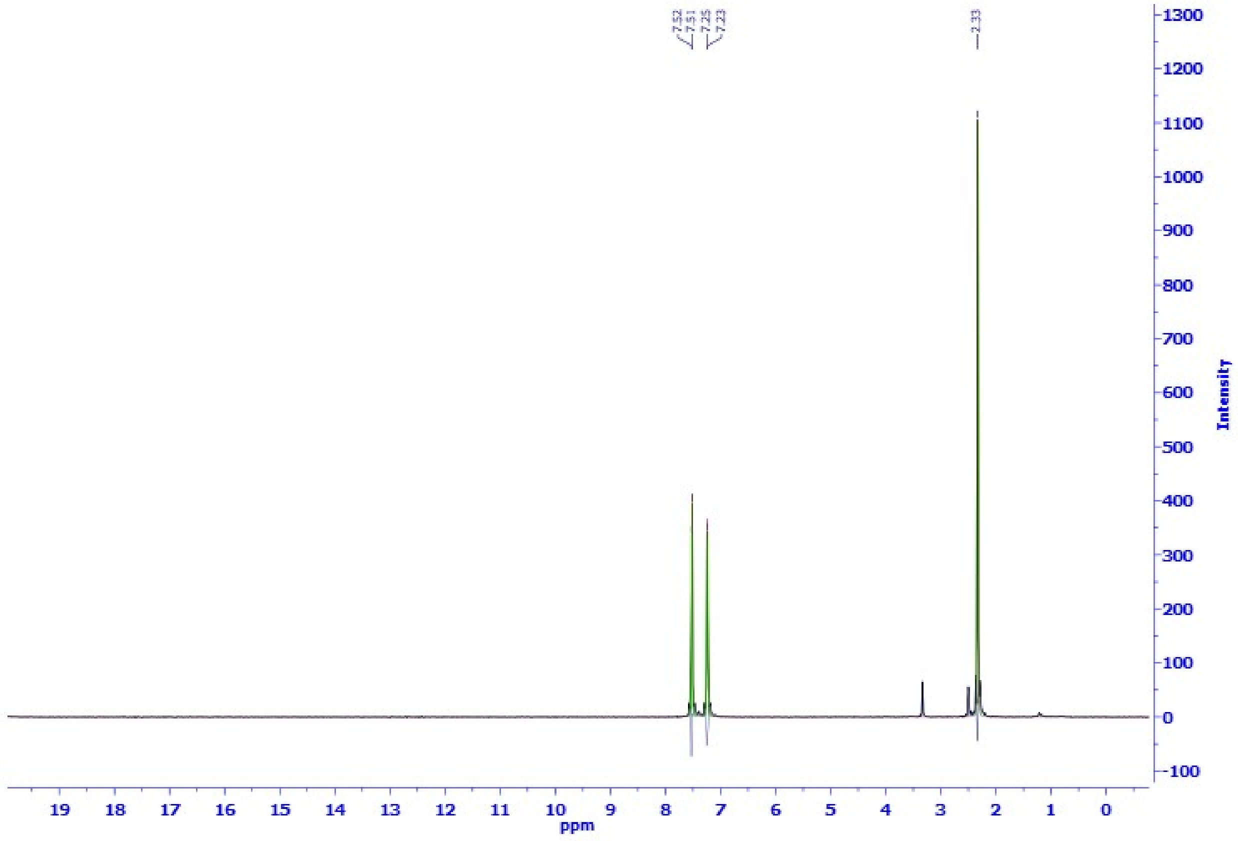

Figure 1. ^{1}HNMR spectrum 4,4-dimethyl biphenyl

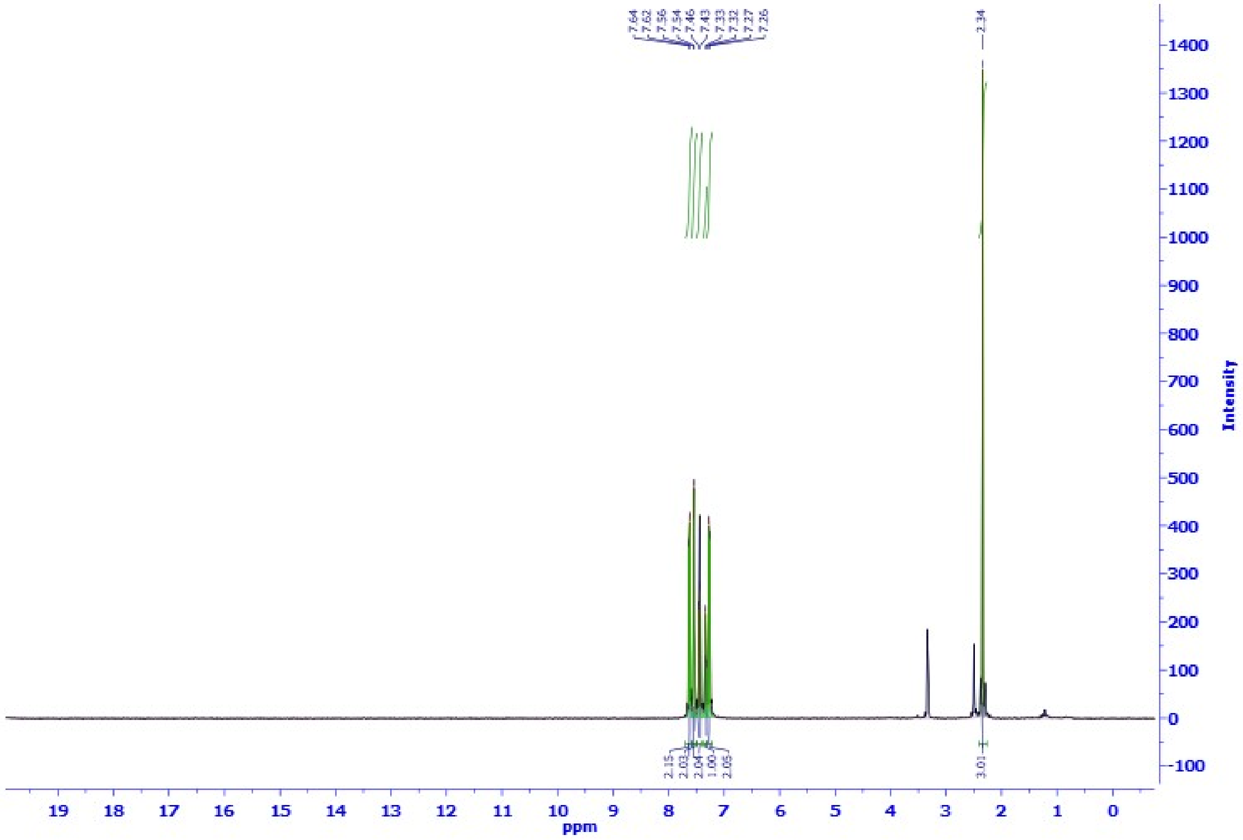

Figure 2. ¹HNMR spectrum 4-Methyl biphenyl

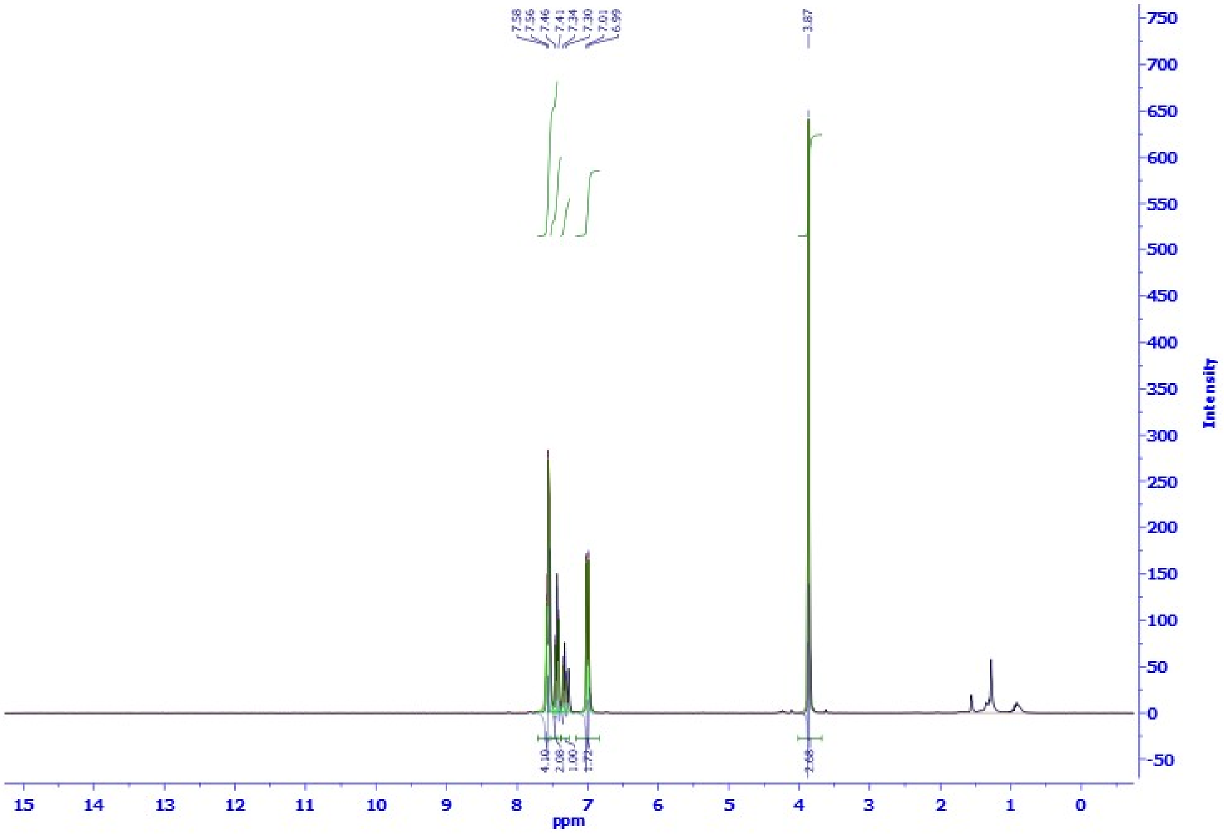

Figure 3. ^{1}HNMR spectrum 4-Methoxy biphenyl

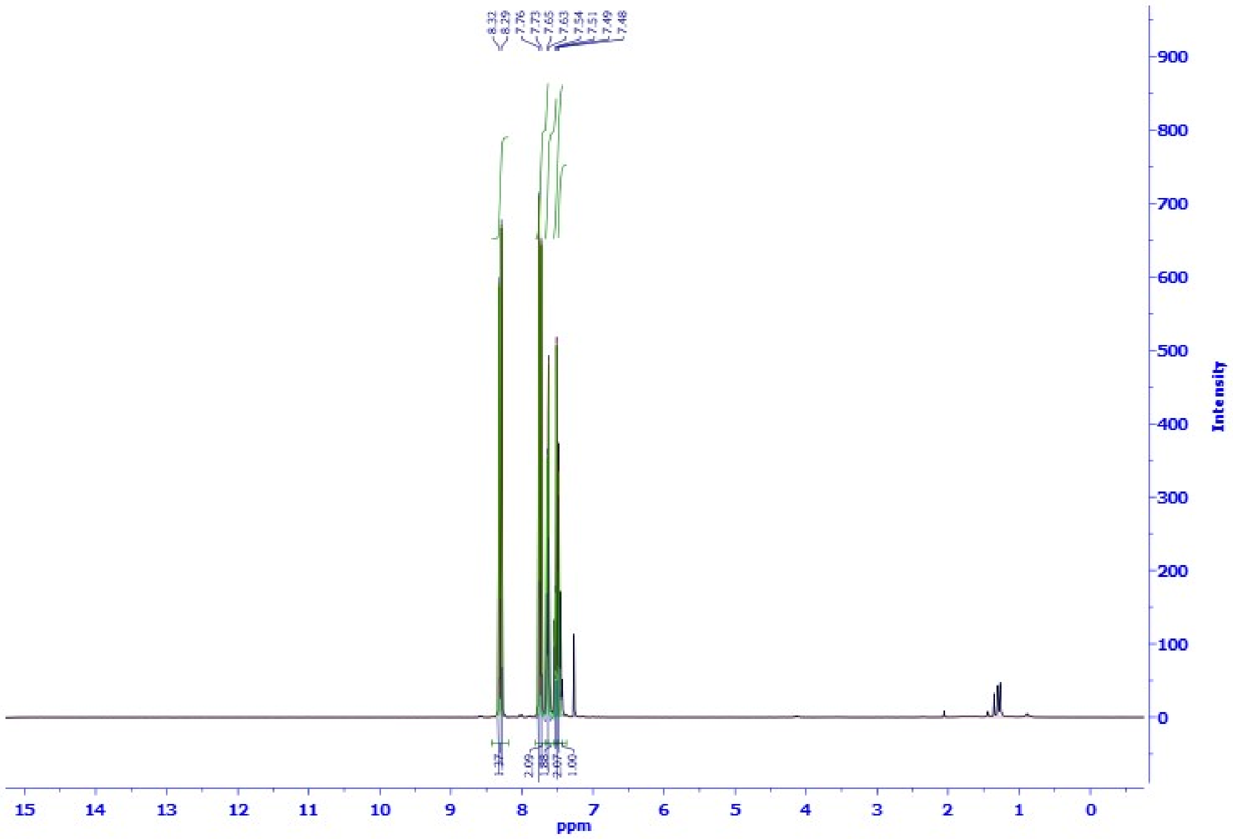

Figure 4. ^{1}HNMR spectrum 4-Nitro biphenyl

^{13}CNMR:

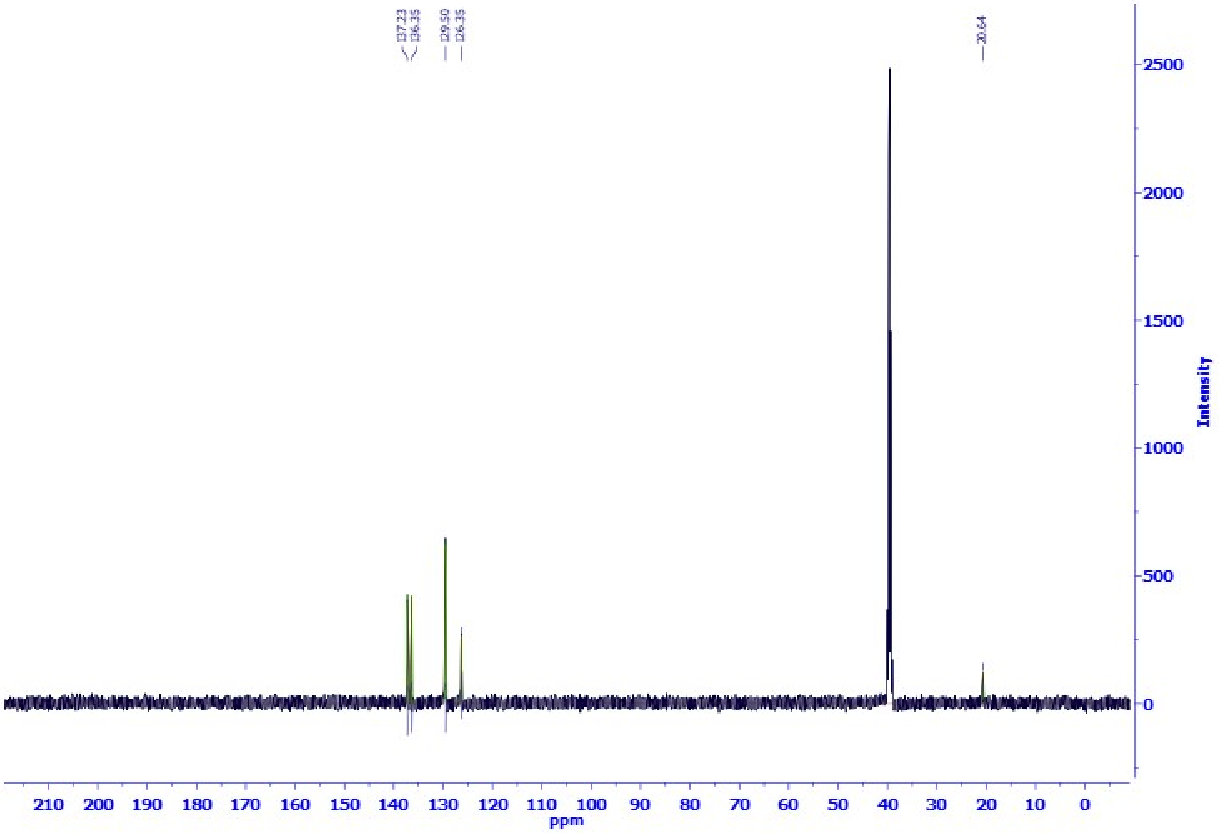

Figure 5. ^{13}CNMR spectrum 4,4-dimethyl biphenyl
